# A Quantitative, Non-Destructive Methodology for Habitat Characterisation and Benthic Monitoring at Offshore Renewable Energy Developments

**DOI:** 10.1371/journal.pone.0014461

**Published:** 2010-12-29

**Authors:** Emma V. Sheehan, Timothy F. Stevens, Martin J. Attrill

**Affiliations:** 1 Peninsula Research Institute for Marine Renewable Energy (PRIMaRE), Marine Institute, University of Plymouth, Plymouth, United Kingdom; 2 Griffith School of Environment and Australian Rivers Institute - Coasts and Estuaries, Griffith University, Gold Coast, Queensland, Australia; Institut Pluridisciplinaire Hubert Curien, France

## Abstract

Following governments' policies to tackle global climate change, the development of offshore renewable energy sites is likely to increase substantially over coming years. All such developments interact with the seabed to some degree and so a key need exists for suitable methodology to monitor the impacts of large-scale Marine Renewable Energy Installations (MREIs). Many of these will be situated on mixed or rocky substrata, where conventional methods to characterise the habitat are unsuitable. Traditional destructive sampling is also inappropriate in conservation terms, particularly as safety zones around (MREIs) could function as Marine Protected Areas, with positive benefits for biodiversity. Here we describe a technique developed to effectively monitor the impact of MREIs and report the results of its field testing, enabling large areas to be surveyed accurately and cost-effectively. The methodology is based on a high-definition video camera, plus LED lights and laser scale markers, mounted on a “flying array” that maintains itself above the seabed grounded by a length of chain, thus causing minimal damage. Samples are taken by slow-speed tows of the gear behind a boat (200 m transects). The HD video and randomly selected frame grabs are analysed to quantify species distribution. The equipment was tested over two years in Lyme Bay, UK (25 m depth), then subsequently successfully deployed in demanding conditions at the deep (>50 m) high-energy Wave Hub site off Cornwall, UK, and a potential tidal stream energy site in Guernsey, Channel Islands (1.5 ms^−1^ current), the first time remote samples from such a habitat have been achieved. The next stage in the monitoring development process is described, involving the use of Remote Operated Vehicles to survey the seabed post-deployment of MREI devices. The complete methodology provides the first quantitative, relatively non-destructive method for monitoring mixed-substrate benthic communities beneath MPAs and MREIs pre- and post-device deployment.

## Introduction

Harnessing renewable energy from the sea is of global importance in the context of both addressing climate change and delivering, for example, the UK Government's target of producing 33 gigawatts of energy from renewable sources by 2020 [1], thus meeting the EU general requirement for 20% of energy to come from such sources by that date. There is a great energy potential in the sea around the UK including wave, tidal and offshore wind – for example, the UK has 40% of Europe's wind resource [2], [3]. When locating technology that can convert renewable energy into electricity it is also a requirement to measure the local and wider environmental impacts that arise from the construction and operation of these devices, so that these developments can be best managed in future when the scale of this industry increases. All of these developments are in contact with the seabed to some degree, whether through concrete piling (e.g. offshore wind turbines), metal structures (e.g. tidal stream turbines such as Strangford Lough, Northern Ireland), or a network of mooring cables and sub-surface electricity hubs necessary for securing and operating wave-energy devices (e.g. the planned Wave Hub development off North Cornwall, SW England).

In the UK, a series of Strategic Environmental Assessments (SEAs) have been undertaken to quantify these key natural resources [4] and, in turn, identify where structures to harness the energy will need to be placed. Often, the seabed habitat at such sites is variable, e.g. a mix of rocky ledges, boulders and soft sediment patches, particularly at high energy tidal stream and wave sites, making standard monitoring methodology difficult to implement. Traditionally, destructive sampling methods (such as grabs, dredges and trawls), or else diver-conducted surveys, have been utilised to determine habitat classification and characterise the benthic community. Whilst useful for relatively small, discrete areas, these methods are impractical for monitoring at the scale of the wind or wave farm because of the prohibitive expense, and/or the damage caused by this type of sampling, which may be detrimental to the environmental aims of the development or inappropriate if conservation considerations need to be taken into account. It has been recognised that many offshore energy developments could potentially act as *de facto* Marine Protected Areas (MPAs) [5], so sensitive methods are required to help boost the environmental credentials of the marine renewable energy sector. Remote sensing, especially acoustic methods such as sidescan or swath mapping, can be used to rapidly characterise large areas of the sea floor [6], and is extremely useful in this respect, but in order to give information about biological distributions, this requires verification from detailed in-situ sampling of components of marine biodiversity [7].

Diver conducted underwater surveys can provide the necessary detailed information [8]; however, these are extremely costly for anything but small areas, and the operation is very complex, particularly in water depths >40 m. The key target areas for wave Marine Renewable Energy Installations (MREIs) will, in most cases, preclude the use of divers: the Wave Hub study area, for instance, covers a footprint in excess of 24 km^2^ including control sites, is >50 m deep, and subject to high wave energies and strong currents. In other industries, (e.g. offshore oil and gas production), remote operated vehicles (ROVs) are used in place of divers for many tasks, including biological surveys [9]. Again, ROVs of the class required to operate in these conditions are expensive, require significant top-side support, and take time to cover large areas of seabed. Increasingly, biological surveys are carried out with relatively inexpensive video sensors mounted on towed sleds [10]–[11]; these are particularly effective on low-relief, soft sediments where large areas can be covered relatively quickly. However, where substrate type comprises mixed or rocky habitats, sled mounted sensors are impractical because of the risk of entanglement, leading to equipment damage or loss, and because heavy sleds are themselves destructive, functioning in a similar manner to a light dredge or trawl.

This paper reports on the successful use of a relatively new methodological design specifically developed for the needs of mixed-habitat offshore areas. In this design, High Definition (HD) camera technology is mounted on a flying array which is towed behind a boat yet is almost a non-contact method of surveying the sea bed, so covers a large area with minimal damage. The design is an enhancement of the method developed for comprehensive surveys of Moreton Bay, Australia [12]–[15], based in turn on the design principles of Barker et al. 1999 [16], but much reduced in size and complexity. Here we describe the design and use of this camera set-up to quantify the benthos over a range of environmental conditions, from shallow water reef habitat types to extreme tidal currents and deeper water with a high wave climate, where it has now been actively tested. The limitations of using a towed array are also discussed and the next stage of this work is proposed which involves ensuring the compatibility and comparability of the use of a HD video camera mounted on a flying array to one mounted on a Remote Operated Vehicle (ROV). We suggest that this is the ideal methodology to employ across future offshore developments.

## Methods

### Survey requirement

This equipment has been developed primarily for the quantification of the sea bed at two contrasting sites in the southwest of the UK: Both sites feature mixed habitat seabed: the Wave Hub site off the north coast (Cornwall), which is extremely exposed and lies in 50–60 m, and the Lyme Bay site off the south coast (Devon/Dorset), which is more sheltered and ≈25 m deep. The Lyme Bay reefs are being monitored to determine the effectiveness of the newly designated 206 km^2^ MPA [17]. Good quality, high resolution images of the seabed are required in order to detect changes to the benthic habitats and communities over time as a result of the Wave Hub construction, whether these are positive or negative [5]. The survey method needs to be non-destructive and able to be deployed on mixed substrates, including moderate relief reef structures, in order to survey high-biodiversity, sensitive reefs such as Lyme Bay. Subsequently, the equipment has been commissioned to attempt to survey an extreme tidal stream area in Guernsey, Channel Islands, (Depth 43–56 m;Current up to 2.6 ms^−1^, though 1.5 ms^−1^ at “slack” water) to assess the technique as a method for characterising the seabed in areas of potential tidal energy generation.

### Flying Array

**Figure 1:**
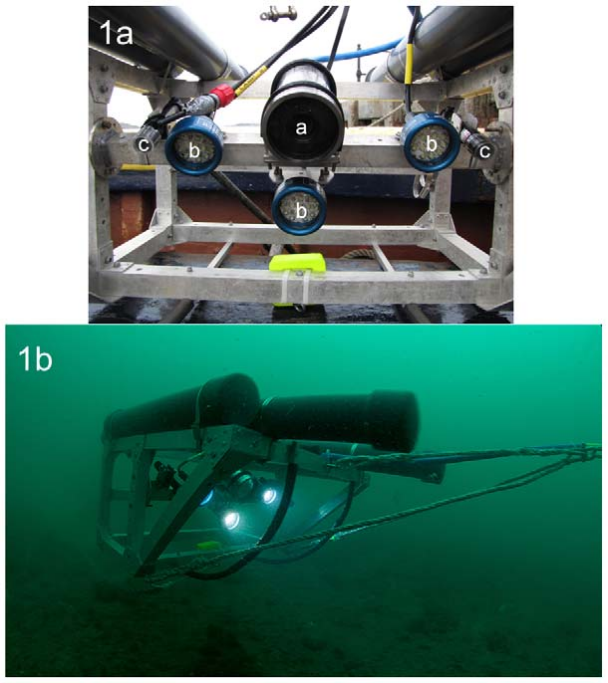
Details of the flying array equipment and operation underwater. 1a. The flying array with High-Definition (HD) video camera attached (labelled “a”). Highlighted on the photo are lights (b), laser pointers (c). The frame dimensions are approximately 1×1×0.5 m (L×W×H). The buoyancy tubes extend approximately 0.25 m beyond the frame fore and aft. 1b. The flying array in operation underwater, illustrating the buoyancy tubes supporting the array attached to the top of the frame.

The aluminium frame of the flying array (Figure 1a) is a modification of the design detailed in Stevens (2003) [12], scaled up 1.5 times to house the extra bulk of the HD video and CTD (Conductivity-Temperature-Depth) equipment. To make the frame neutrally buoyant, ballast tubes of high-strength plastic were attached to the top of the frame (Figure 1b), and calibrated so that the frame was just positively buoyant with all equipment fitted, but without the drag chain (see below). The frame is towed by a floating bridle attached at the two lower front corners and centrally on the upper front panel cross member. It was configured so that the array lifted up slightly as it was towed along.

**Figure 2:**
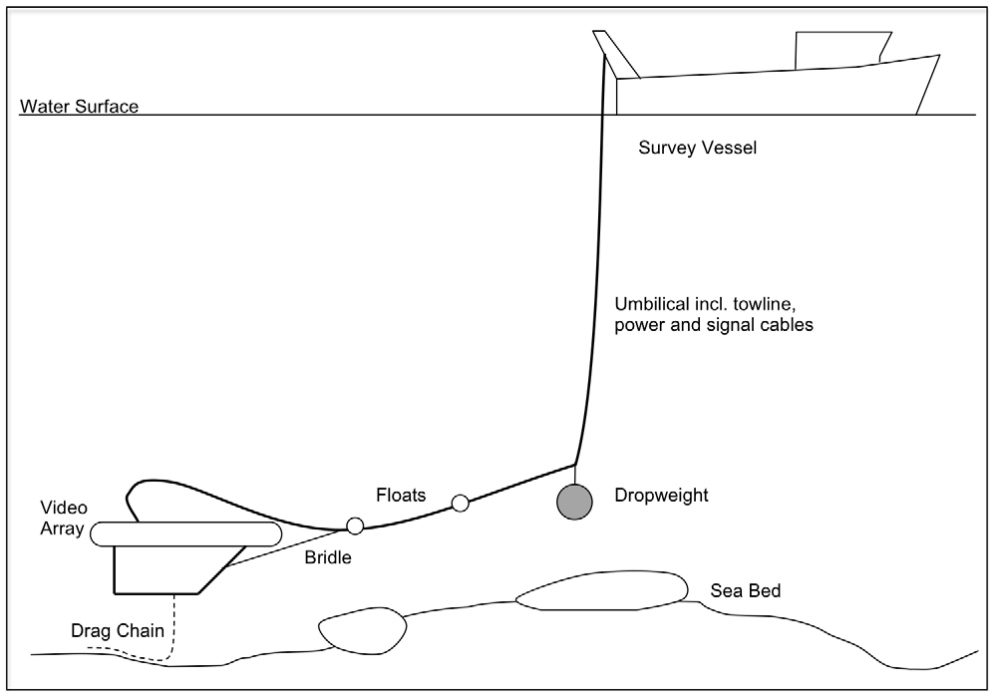
Arrangement for deployment of the video array. Stylised diagram illustrating the full arrangement of equipment during deployment of the video array (not to scale).

A short length of chain extending from the base of the array allows it to “fly” at a predetermined height above the sea bed; this chain is the only piece of the device making contact with the seabed (Figure 2). When the chain is partly on the bottom the array is neutrally buoyant. Changes in bottom topography result in less or more of the chain off the bottom; the array adjusts its height until equilibrium is again achieved. The desired height above the bottom can be adjusted by changing the weight of chain used, or the length of a lightweight rope attaching the chain to the array. This rope functions as a weak link, breaking if the chain snags on the sea bed to allow recovery of the array and avoid damage to the umbilical. The weight of chain used is dependent on the environmental conditions, especially current and wave surge. In fair conditions, such as Lyme Bay, 8 mm stainless steel chain can be used (Length: 3.15 m, Width: 12 mm, Weight 10 kg); in more extreme, high-wave conditions, a shorter thicker chain is more suitable, the compromise being the slightly increased impact that sampling has on the sea bed. This is still, however, minimal compared with traditional equipment.

### Cameras, lights and lasers

A HD video camera, lights, lasers, and a CTD (mini CTD profiler: Valeport Ltd) were mounted on the array, and could all be accessed in real time from the surface. Digital video cameras have been regularly used in recent years to capture larger, less abundant mobile fauna such as fishes and crustaceans [18], with separate stills cameras employed to obtain images for quantification, e.g. for abundant sessile fauna such as sponges and corals. Frame grabs from digital video are of limited quality, so that smaller, or more cryptic, species may not be identified to species level; larger less common species may be missed entirely if only stills images, which represent a subsample of the video track, were relied on to capture them. The decision was therefore made to test the use of a single HD video camera to achieve both sets of samples, the device chosen being a Surveyor-HD-J12 colour zoom titanium camera, 6000 m depth rated, 1080i/720p. This camera has performed well at a range of depths and conditions, allowing operators to zoom, change the aperture and select 720p or 1080i. We have it set at 720p as 1080i would be more prone to blurring. In 1080i, only half of the lines are displayed in each frame, but when viewed at normal speed the human eye integrates successive frames to perceive excellent quality video; however, when viewing individual frames for data extraction, quality is reduced. In contrast, in 720p there are fewer lines, but all are present in each frame and the quality for frame by frame data extraction is superior. The optical zoom is 10∶1 (5.1 mm to 51.0 mm focal length), with an additional 4x digital zoon that is deactivated as standard. The angle of view is 61° diagonal in air and 45° diagonal in water when the camera is set at its optimum elevation angle of 100°; the window is made of sapphire glass. Focus can be controlled from the front window to infinity. The umbilical was connected topside to a Bowtech System power supply/control unit, which allowed control of the camera, focus, zoom and aperture, and intensity of each light.

HD video requires good illumination to make the best use of the resolution available. LED lights were selected (Bowtech Products limited, LED-1600-13, 1600 Lumen underwater LED) as they last for thousands of hours, are robust and will not burn out if left on. Incandescent lights require greater power, are prone to breaking, the bulbs need replacing often and can be dangerous due to quick over-heating on board a boat if accidentally left on. The camera produced good quality images when all three lights were used, and at speeds over the ground of up to 0.25 ms^−1^ where high quality frame grabs can still be extracted.

Lasers were mounted onto the flying array to standardise the field of view, a necessary requirement in order to make species counts quantifiable. The lasers were set a fixed distance apart and parallel, so that the two points were always 50 cm apart; the width of benthic habitat captured on a standard image is 60 cm, which can be increased with height above the sea bed. When sampling in poor water visibility, to improve the picture quality so that species identifications could still be made with confidence, it was necessary to reduce the distance between the lasers to 30 cm and fly the array closer to the seabed. The apparent distance between the laser dots on the image is therefore an indication of how far above the substrate the camera is flying; all frames and videos can therefore be scaled to allow quantification of densities, and images that are outside the required tolerances can be discarded. We trialled both red and green lasers; while both were adequate, green lasers (532 nm) were far more prominent on both the video and still images. The units we used (Beam of light technologies, Inc: Scuba-1 Underwater dive laser) have a robust housing which was mounted onto the array using a custom made aluminium bracket and secured with cable ties. The full weight of the flying array with equipment attached was 30 kg. Camera, lights, CTD and topside computer were powered by a portable 2KVA Honda Generator through a 1000VA UPS (Uninterrupted Power Supply), which delivered adequate power with more than 30 minutes managed shutdown in the event of generator failure.

### Configuration and operation

The array was tethered to the vessel by a single umbilical consisting of a c.17 mm diameter reinforced tow cable rated for 140 kg load, and incorporating 22 cores for power to the camera, lights and CTD, and to return the video and CTD data signals to the surface. The array can be deployed from a small vessel in the arrangement shown in Figure 2. To reduce strain on the cable a 14 mm tow rope was used to take the strain of the flying array and the drop weight. The umbilical was secured to the tow rope using cable ties and duct tape between the flying array and the drop weight. From the drop weight, the tow rope and umbilical were independent of each other, so that the tow weight could be hauled using a winch or pot hauler, and the umbilical was manually hauled. The drop weight was attached to the tow rope approximately 10 m ahead of the array. This ensures that the umbilical between the ship and the drop weight remains close to vertical, keeping the array within about 15 m horizontally of the vessel, minimising error in GPS derived positional information. The drop weight also acts to dampen pitching and snatch of the array from swells at the surface and was attached with a weak link to prevent losing the array if snagged. Small floats (2×850 gram) were attached to the umbilical between the array and drop weight to prevent the umbilical contacting the seabed when the strain is off. Control of the speed of the array over the bottom in critical: it must be limited to 0.25 ms^−1^ (c. 0.5 knots) or less or the video imagery is blurred and unusable for quantitative analysis. This arrangement has been successfully used in a wide range of substrate types. Tow lengths were nominally 200 m, requiring the array to be on the bottom for approximately 20 minutes per site. The HD video stream was recorded with no downscaling using a 3-Dive HD-DVR light recorder, which allowed real-time viewing of the video footage (essential to control the deployment of the array) and overlaid date, time and mission number onto the video stream. GPS derived positional information can also be overlaid. Video footage was backed up daily onto a 1 terabyte hard drive.

### Extracting quantitative data

To use video imagery in a quantitative way, several attributes are needed [10], [19]. The field of view of the camera must be accurately calibrated and the total area, or distance, of each transect must be accurately recorded. The locations sampled must also be accurately known, relative to the scale of the survey, to allow spatial analysis. Data should be extracted in a numerical form to allow quantitative statistical analysis.

In this study, the field of view was calibrated by flying the array over a known grid, and then calculating a scaling factor based on the position of the laser dots on the video image. The distance of each transect, and hence the area sampled by a known track width (the distance between the laser dots), was calculated from GPS positions taken at the beginning and end of each transect.

**Figure 3:**
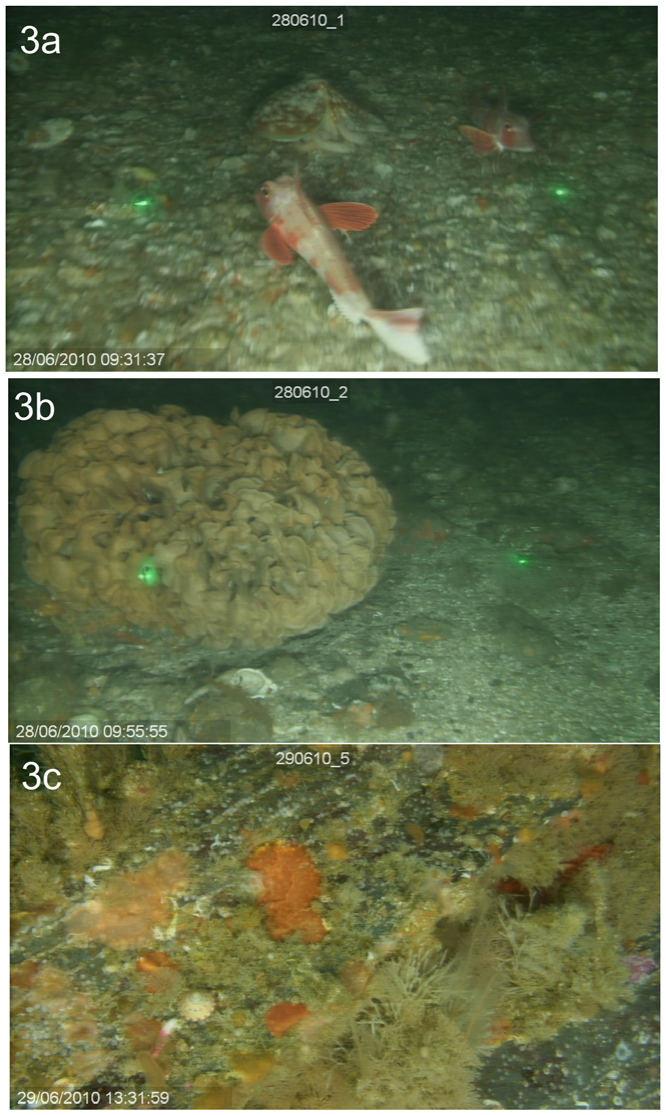
Example frame grabs from the High Definition video. Example frame grabs from the HD video used for quantitative assessment of habitat characteristics or organism assemblage composition, from Wave Hub site, Cornwall UK, at 60 m. 3a. Two Red Gurnard (*Trigla lucerna*) and a lesser octopus (*Eledone cirrhosa*, centre back). 3b. A large Ross Coral (*Pentapora fascialis*, a bryozoan). In both images, the distance between laser points is 50 cm. 3c. One of 100 random frame grabs/transect for detailed assemblage composition assessment illustrating high resolution enabling identification of smaller organisms.

Quantitative biological information was extracted in two ways. Large, obvious elements of the epibenthos were counted by viewing the video at normal speed, and recording each identifiable organism as it passed through the “gate” formed by the two laser dots (Figure 3a,b). This raw count was converted into density (individuals m^−2^) by dividing by the calculated area sampled. This allows a rapid derivation of the quantitative information on the gross elements of the macro-epibenthos. Detailed information on either density or percent cover of smaller organisms, including metrics of infaunal density and bioturbation such as burrow densities, was derived from the high quality still images from random frame grabs (e.g. Figure 3c). Nominally, 100 frames were randomly selected from each transect; those that were blurred by excessive camera movement, or where the bottom was otherwise obscured, were discarded. The remainder were examined frame-by-frame and the number of each identifiable organism recorded. The results of this biological survey will be reported elsewhere, but the method employed allowed a rich quantitative dataset to be collected cost-effectively, in conditions that precluded the use of other methods. Further work has been carried out to develop methods of accurately measuring the size of organisms from the video imagery with reference to the laser dots, and will be published subsequently (Coram et al, in prep).

## Results

### Equipment deployment and modifications to operation

The initial set up of the equipment was first tested over several weeks within Lyme Bay during September and October of 2008, where it performed exceptionally well over mixed and rocky ground at depths of around 25 m. This allowed us to move to deploying the flying array at the much more extreme conditions at the Wave Hub site (>50 m depth, 16 km offshore) during June 2009 and 2010 in order to obtain two years' background data prior to construction of the wave energy device testing facility. The planned location of each transect was based on existing data sets, e.g. bathymetry, habitat type, fishing effort, depending on what was important. Transects can be georeferenced using the boats' GPS, hand held devices or using devices which are logged as part of the onscreen overlay, so that they can be resurveyed for the purpose of monitoring within the summed error of the GPS accuracy and the length of the tow.

**Figure 4:**
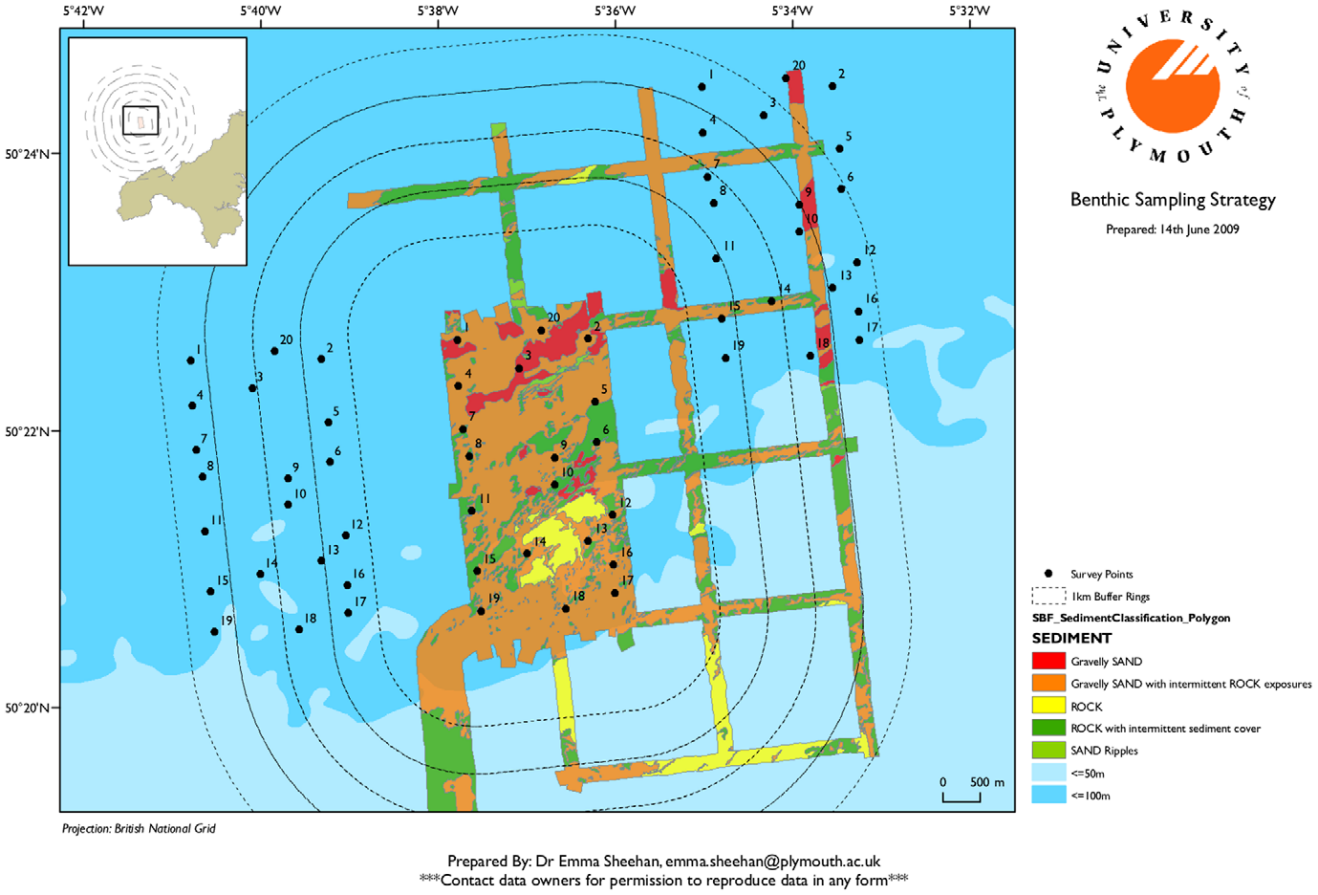
Sampling design at the Wave Hub site, Cornwall, UK. Sampling design at the Wave Hub site, Cornwall, UK, for the High Definition video survey, indicating replicate sample points within the Wave Hub safety zone, plus two control areas. The mixed-nature of the seabed is also highlighted, meaning such sites are impossible to adequately sample using most standard methodology.

Twenty replicate transects were successfully sampled within the Wave Hub site (Figure 4), together with an equal number of samples obtained from two adjacent control areas, allowing a clear framework for assessing impacts within the Wave Hub 8 km^2^ planned safety zone. A total of 60 sites were sampled at an average of 8 transects per day, resulting in high quality video transects and frame grabs suitable for quantitative analysis (Figure 3). The survey vessels were a 12 m fishing trawler and a 10 m Gemini catamaran. The array was deployed over the stern of the boat (taking care to ensure that the cable was kept away from the propeller), and retrieved the same way using a hydraulic pot-hauler or winch.

Throughout the field program the gear performed well in, at times, challenging conditions, demonstrating its adaptability for use in varying situations, with a few modifications required to allow successful operation in the exposed, deep conditions at Wave Hub. During periods of low visibility, image improvement was achieved by reducing lights to minimise back scatter; the lights could also be re-positioned to reduce light reflecting off plankton in the water column. In poor visibility, image quality can be improved by configuring the array so that the camera is closer to the sea bed (by reducing the length of the rope leader); in this case the speed over the ground must also be reduced to avoid blurring. At the Wave Hub site, the typical current on a neap tide was 0.25 ms^−1^ (c. 0.5 knots), which provided an ideal speed to drift with the current. As the current increased, engine inputs were required to slow the rate of drift. At the maximum current speed that was encountered, 1 ms^−1^ (c. 2 knots), the 23 kg drop weight was replaced with a 31 kg weight to hold the umbilical close to vertical. In rougher conditions, performance was improved by the use of a heavier drag chain (9 kg: 4 m×35 mm) to decrease the buoyancy compensation response time. These modifications allowed the array to perform as well as in shallow calmer water.

Following the success of the array at the Wave Hub site, methods of deployment were further developed to characterise the seabed in stronger tidal stream conditions, east of Guernsey (Channel Islands) between the islands of Herm and Sark. The site, known as ‘The Big Russell’ (49° 27′ N, 2° 25 W) has been identified as a suitable location for a tidal MREIs, with tidal currents of up to 2.6 ms^−1^. The channel is approximately 50 m deep and has a seabed with mixed habitat types comprised of patches of shells, gravel, slate slabs, boulders, and steep rocky reefs.

A 14 m fishing trawler was the research platform and the equipment was deployed from an over-head winch. The configuration of the drop-weight and chain was adjusted to current velocities of up to 1.4 ms^−1^. A heavier drop weight (52 kg) was deployed and a heavier, larger chain was attached to the array (13.5 kg: 4 m×40 mm). The added chain does add impact to the seabed, but was unavoidable in the conditions. Also, the arrangement of the boat and array was modified. The current and prevailing wind typically moved the boat faster than the array needed to be towed, as with the Wave Hub example, and so the boat was driven into the current. The boat can also be driven astern (in reverse) but that becomes risky as the array will fly under the boat and the umbilical could get caught in the boat's propellers. Better results were achieved when the boat was driven at approximately a 45° angle into/across the prevailing conditions, so that the boat still moved backwards but at the desired speed.

It was often not possible to work throughout the tidal cycle at the Guernsey site, and so survey effort was focused around the 2 periods of slack water per day (1.5 ms^−1^). In 10 working days, it was still possible to achieve 76 useable 200 m tows across the full range of habitat types previously discussed, dispersed between sites identified as possible sites to be developed and sites which would be suitable controls.

### Sample quality and data analysis

**Figure 5:**
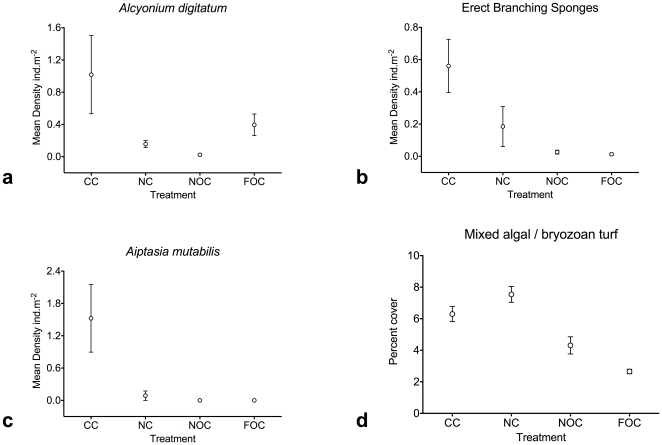
Example data analysis from HD video transects and frame grabs. Analysis of example data for four taxa from the Lyme Bay MPA study highlighting the potential for the video methodology to provide robust data for testing hypotheses. Here treatments are: existing voluntary closures to fishing (CC), new enforced closures (NC) and near or far controls where fishing continues (NOC, FOC). Data represent baseline conditions in September 2008. 5a, b: data from analysing whole replicate transects; 5c,d: data from detailed random frame grabs (n = 30/transect).

At all three survey sites, the methodology has obtained robust, replicate samples (200 m video transects; Figure 3a,b) which can be analysed to characterise the habitat and provide quantitative data on large organism distribution. Where different treatments exist, these data can be analysed statistically (e.g. ANOVA, PERMANOVA) to determine significance of differences between treatments (e.g. MPA areas vs controls, Figure 5a,b). From each transect, random frame grabs (Figure 3c) provide samples for more detailed analysis of benthic assemblages and smaller organisms, allowing additional analysis to support testable hypotheses (Figure 5c,d).

## Discussion

### Outcomes of this study

The methodology described here is ideal for characterising and sampling large areas comparatively quickly and thus cost-effectively, yet without damaging the environment. The full survey to provide background information at Wave Hub obtained samples each year to be representative of a total area of 24 km^2^, yet the actual physical sampling only took 8 days, excluding days lost to bad weather. During this time, 60×200 m long transects were sampled over mixed ground at depths >50 m, from which robust data can be extracted at the species level where required. It is unlikely that any other method could have so adequately characterised the mixed substratum seabed at this site with similar effort. During the time at sea, the flying array was used also to take regular samples along the full length of the cable route from the coast to the Wave Hub site (6 transects along 22 km) in order to assess any change following the laying of the cable. It is therefore feasible that the camera equipment can also be used to visualise cables once laid in order to assess their condition and integrity.

The site off Guernsey was even more demanding, not only being deep (up to 56 m) but also with extremely strong tidal stream currents (up to 2.6 ms^−1^) that make this area so suitable for energy generation, yet so difficult to sample, particularly as the current speed tends to result in hard, or at least mixed, substrata. However, if habitat characterisation and impact assessments are to be undertaken to support renewable energy development in tidal races, quantitative and repeatable sampling is essential. To deal with this environment the gear needed some modification coupled with skilled boat handling to enable the array to fly at a suitable speed to record quality video suitable for analysis. Despite the restrictions due to sampling over slack water, over 70 complete 200 m transects were obtained with enough replication and detail to characterise the seabed and act as a baseline for future development. As far as we are aware, this is the first time quantitative remote samples have been successfully obtained from such a tidal stream site, highlighting the potential of this methodology as a solution for monitoring in such environments.

The relative performance of remote video versus diver census in surveying mixed seabed habitats was not investigated in this study, since video was clearly the only method that would permit the study to proceed on the scale desired at depths >50 m at Wave Hub and Guernsey (particularly when coupled with high tidal stream). Cailliet *et al.* [20], however, compared quantitative sampling from trawl, video sled and ROV and concluded that the video sled was less subject to gear avoidance and provided more accurate estimates of density. The flying arrays used in this study and others [21] have the additional advantage of being useable on almost any substrate, with very low impact (unlike sleds and trawls). During operation in Lyme Bay, divers were deployed to observe the movement of the array across the bottom and the impact of the chain, the only point of contact with the seabed. Overall the chain has minimal effect, dragging across objects. On rare occasions, at a particular angle of impact, the chain can bend tall flexible structures, but no permanent damage was observed and impact is minimal compared with a full benthic camera sled on the sea bed. The chain does leave a trail in soft sediment, but that seems to be the only notable impact. Stevens and Connolly [15] commented on the cost-effectiveness of the flying array video method (the precursor of the design described here) and concluded that it offered an order of magnitude cost-saving over conventional methods, as a result of the larger area covered per fieldwork day, lower crewing requirements, and the smaller survey vessel required. In this study, for the same reasons, we suggest that no other method would allow a comparable survey for less than 3 to 5 times the cost. We acknowledge, as have others [11], [22], that there is a clear trade-off in using video as the primary data-collecting source, in that taxonomic resolution can be lower (depending on target organisms) because specimens are not retrieved to verify identification, and especially in the case of smaller taxa, identification may be limited to higher taxonomic levels. Nonetheless, the method allows repeatable, quantitative surveys over sufficiently large areas to meet the needs of long term monitoring and the continued development of HD technology is making detailed identification from video frame grabs more accurate.

### Problems and limitations

The equipment has successfully sampled shallow rocky and mixed habitats with high conservation status in Lyme Bay, a deep, high-energy site 16 km offshore and a deep, high-current coastal site. A key to the success of the method is effective control of speed over the ground (≤0.5 knots, 0.25 m/sec) in order to take HD video for clear frame grabs. Problems potentially arise, therefore, where high tidal streams exist if it is not possible to control the speed of the array; useful video is possible at speed, but not satisfactorily clear frame grabs for quantitative species analysis at this level. At the Wave Hub site, currents were reaching 1 ms^−1^ (2 knots) at maximum tidal flow, yet successful samples at lower speed were possible by using the engine to slow the rate of drift. Furthermore, by experimenting with the alignment of the vessel to the current at the tidal stream site in Guernsey, it was possible to achieve sampling at currents up to 1.5 ms^-1^, this time by steaming into the current at an appropriate angle, allowing the vessel to drift. The extra weight of chain used held back the array in the current and so, in this instance, everything moved backwards at a suitable slow speed (boat, drop weight and array). Appropriate ship-handling to manage speed over the ground will be particular to each vessel used; more sophisticated technology (e.g. variable pitch propellers) may aid in this.

A further problem faced by traditional survey methods is that sampling will be difficult when, for example, wave farms are fully operational and devices are deployed over the sea surface with a set of subsea moorings and cables. Once safety zones are in place around such sites (e.g. Wave Hub), sampling under devices will not be possible using traditional environmental survey methods. Similarly, the towed camera device will also not be suitable for surveying the seabed close to large, moving wave energy converters, but the data gathering method through HD video means that the same equipment can be fitted to a suitably large and powerful Remotely Operated Vehicle (ROV) which can then be used for small-scale sampling in the vicinity of such MREIs to re-survey areas previously sampled using the towed array. Programmes of device comparison are currently underway (using a Saab SeaEye Falcon ROV) to make sure samples taken using the flying array and ROVs are standardised and comparable, giving the complete monitoring package pre- and post-device deployment. Whilst the use of an ROV will be essential when sampling close to, or under, devices, the flying array method can continue to monitor control areas cost-effectively and relatively quickly (e.g. the two control areas either side of Wave Hub in Figure 4).
